# Exploring racial and gender disparities in voice biometrics

**DOI:** 10.1038/s41598-022-06673-y

**Published:** 2022-03-08

**Authors:** Xingyu Chen, Zhengxiong Li, Srirangaraj Setlur, Wenyao Xu

**Affiliations:** 1 CSE, University of Colorado Denver, Denver, 80204 USA; 2 CSE, University at Buffalo, SUNY, Buffalo, 14228 USA

**Keywords:** Computational science, Computer science

## Abstract

Systemic inequity in biometrics systems based on racial and gender disparities has received a lot of attention recently. These disparities have been explored in existing biometrics systems such as facial biometrics (identifying individuals based on facial attributes). However, such ethical issues remain largely unexplored in voice biometric systems that are very popular and extensively used globally. Using a corpus of non-speech voice records featuring a diverse group of 300 speakers by race (75 each from White, Black, Asian, and Latinx subgroups) and gender (150 each from female and male subgroups), we explore and reveal that racial subgroup has a similar voice characteristic and gender subgroup has a significant different voice characteristic. Moreover, non-negligible racial and gender disparities exist in speaker identification accuracy by analyzing the performance of one commercial product and five research products. The average accuracy for Latinxs can be 12% lower than Whites (p < 0.05, 95% CI 1.58%, 14.15%) and can be significantly higher for female speakers than males (3.67% higher, p < 0.05, 95% CI 1.23%, 11.57%). We further discover that racial disparities primarily result from the neural network-based feature extraction within the voice biometric product and gender disparities primarily due to both voice inherent characteristic difference and neural network-based feature extraction. Finally, we point out strategies (e.g., feature extraction optimization) to incorporate fairness and inclusive consideration in biometrics technology.

## Introduction

Demographic inequity, like racial and gender disparities in biometric systems, has received significant attention in recent years. There are rising concerns about whether significant differences exist between the performance of the biometric system on subgroups, thereby privileging and disadvantaging specific subgroups. Previous studies have shown that such disparities exist in facial biometrics^1^. In contrast, racial and gender disparities remain unexplored for voice biometrics. Voice biometrics are extensively used in critical biometric systems worldwide in applications related to public services such as online banking^2^, access control^3,4^, healthcare^5^, and smart home technologies^6^. Voice biometrics is a technology that utilizes the recognition of voice patterns to identify individuals. As a practical behavioral biometrics modality, voice biometrics offers many benefits in terms of security, user-friendliness, low cost, and high social acceptance.

**Figure 1:**
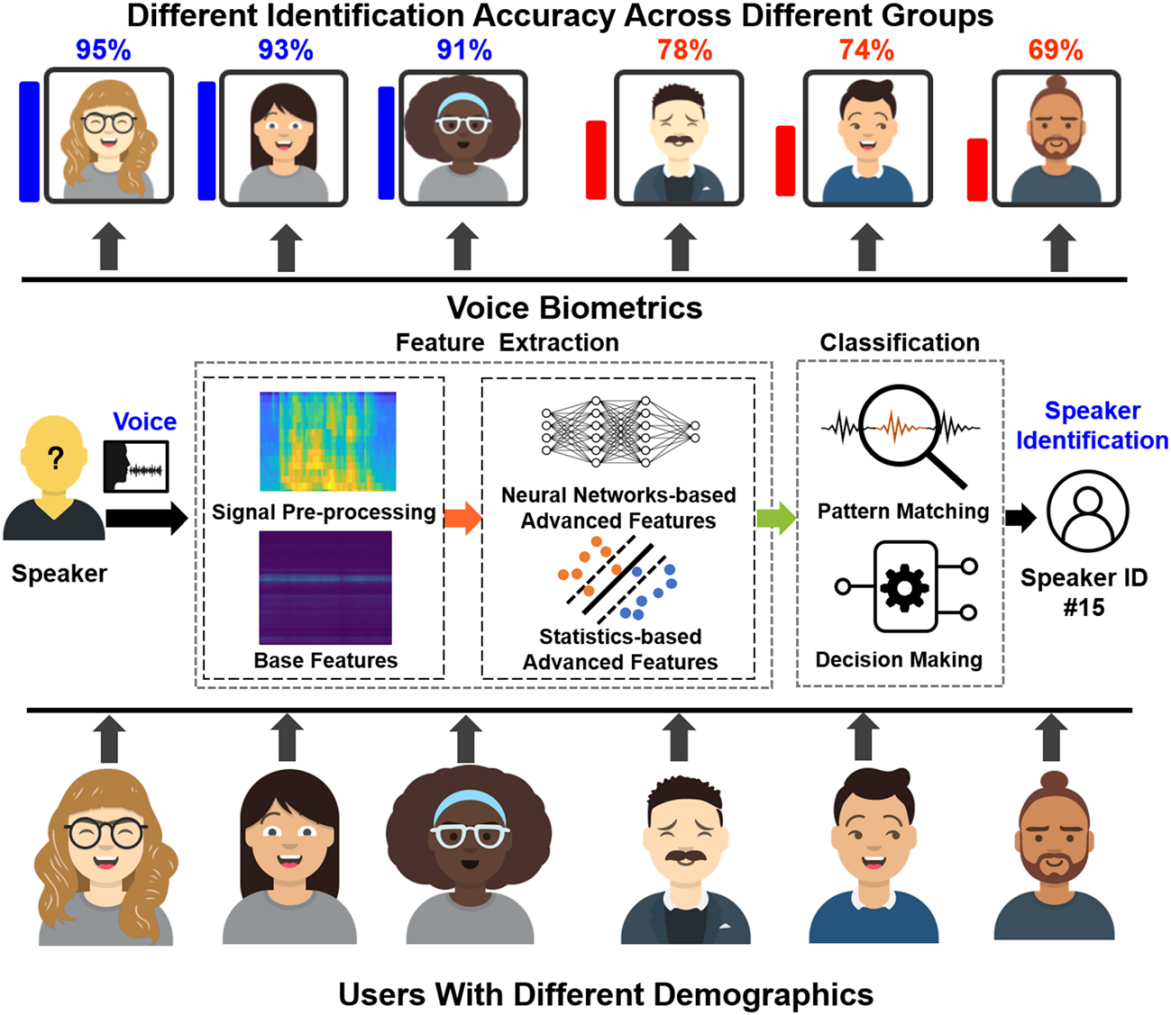
These voice biometric services could produce significantly different identification results towards speakers with diverse demographic backgrounds.

However, given the increasing concerns about potential demographic biases in biometrics in general, it is critical to examine whether racial and gender disparities exist in voice biometrics as well and if so, to what extent. Previous explorations have demonstrated that disparities exist in other voice-based systems such as automatic speech recognition^7^. Given that racial and gender differences have been documented in voice inherent characteristics^8^, these differences perhaps affect the performance of voice biometrics on users with different demographic backgrounds. These racial or gender disparities can result in crucial bias issues or other social problems when voice-based systems are deployed on a large scale. Therefore, we aim to explore if the racial and gender subgroups have different voice inherent characteristics and then cause disparities in voice biometric performance, as shown in Fig. 1. To achieve this goal, there are two main challenges.

*(1). What are the differences among voice inherent characteristics among racial and gender subgroups, and how to reveal these differences?*

To evaluate the voice inherent characteristics under demographic factors (racial and gender), we investigate the essential voice properties for each race and gender of the voices in our matched datasets regarding 15 representative fundamental voice metrics: Formants Frequency^9^, Mel Frequency Cepstral Coefficients (MFCC)^10^, Pitch onsets^11^, Root Mean Square (RMS)^12^, Roll-Off^13^, Centroid^14^, Spectral entropy^15^, PDF entropy^16^, Permutation entropy^17^, and SVD entropy^18^. These fundamental metrics represent the essential and primary characteristics of the voice, which are also the base for the voice biometrics system (see details in “Voice fundamental metrics” section). Additionally, the matched dataset means the data samples in the dataset are paired up so that speakers in different subgroups share similar characteristics except for the one factor under investigation, which controls for the effects of other “unwanted” factors and is better to explore the racial and gender disparities in voice biometrics.

Since the human voice is a complex signal containing both the speaker’s identity and the linguistic message^19^, to minimize the impacts from these linguistic and accent factors in voice, our analysis utilizes the non-speech voice snippets from the mPower dataset, a clinical observational study purely through a smartphone app interface^20^. In these non-speech voice snippets, the voice activity recorded participants’ sustained phonation by instructing them to say ‘Aaaaah’ into the microphone at a steady volume for up to 10 s. Our study is based on non-speech voice snippets that exclusively contain the genuine and clear ‘Aaaaah’ voice and are 5–10 s long. It is worth mentioning that /ɑ/ (‘a’) is a vowel that can be continuously vocalized and has the most occurrence compared to other syllables^21^. Therefore, the ‘Aaaaah’ voice snippet is feasible and adequate for voice biometrics. Additionally, the voice biometric-based on the short utterance (a spoken word or vocal sound)^22^ is practical and has high user acceptance in real applications^21^. Besides, to preclude the interference of imbalance class in data samples and better explore disparities in voice biometrics itself, we prevent this concern by setting two matched datasets based on race and gender. There are four sub-groups in the matched dataset on race (i.e., four major races in the US^23^): White/Caucasian, Black/African, East/South Asian, and Latinx/Hispanic, noted as White, Black, Asian, and Latinx respectively hereafter. Totally 300 different speakers are randomly collected after selection, 75 speakers for each sub-group, with identical gender distribution. In the matched dataset on gender, there are two sub-groups, female and male. Totally 300 different speakers are selected, including 150 female speakers and 150 male speakers, with an identical racial distribution. (Details can be found in the “Matched dataset” section.) Thus, in this way, we reveal the disparities in voice inherent characteristics among racial and gender subgroups and the corresponding disparity degree.

*(2). What are the differences in voice biometric performance, and how to identify/track these differences source?*

To continue exploring the effect of demographic factors on voice biometrics, we assess racial and gender disparities comprehensively and figure out the underlying source of these disparities, with one publicly accessible commercial product (i.e., Microsoft Azure^24^) and five open-source research products on voice biometrics. 1d-CNN^25^ and TDNN^26,27^ can accomplish 98% and 87% accuracy on the LibriSpeech^28^ dataset. Besides, ResNet-18^22,29^, ResNet-34^22,30^, and AutoSpeech^22^ can achieve up to 79.48%, 81.34%, 87.66% accuracy on VoxCeleb1 dataset^31^. These research products are based on different typical deep learning methods (e.g., feature extractions and network blocks) as illustrated in Table 2. These state-of-the-art voice biometric models achieve the best performance in speaker identification with different representative technologies or support numerous practical voice biometric applications. The speaker identification task is to identify a person from his/her voice. Voice biometric models are multi-class classifications that take the audio of the speaker as input and output the identity of the speaker. Specifically, open-sources models in this work are 300-class-classification, each class corresponding to a speaker identity. The disparities are measured via ANOVA and Kruskal–Wallis tests on voice fundamental metrics and voice biometric performance (i.e., identification accuracy) (detailed in “Statistical analysis” section). Subsequently, our system combined with matched dataset and statistical analysis protocols can be used as a tool to evaluate the fairness of various voice biometrics products.

## Methods

In this chapter, we illustrate our matched datasets, statistical analysis methods, voice characteristic measurements, and the voice biometric models. All methods and experimental protocols were carried out in accordance with relevant guidelines and regulations and were approved by University at Buffalo Institutional Review Boards (IRB) and informed consent was obtained from all subjects or their legal guardian(s).

### Matched dataset

The data used in this work is a subset of mPower-a smartphone-based clinical observational study purely through a smartphone app interface^20^. The voice recording methodology is significantly close to the real practice condition in voice biometrics. Vocal data contains many audio recordings of participants saying ‘Aaaaah’ for 10 s (hereafter called the snippet). The data is labeled with demographic information such as race and gender. To ensure the data’s quality, we manually and carefully checked each recording snippet and eliminated voice snippets with excessive background noise, not recording text as required, or insufficient length. To better reflect the situation in the real world, we employ both healthy participants and participants with diseases. Some diseases have strong evidence showing not correlated with the human voice (e.g., vocal cord, vocal tract, and articulation). Thus, we only keep participants with vocal, bronchial, and lung disease-based diseases that potentially affect the voice (e.g., Asthma, Pneumonia, Bronchitis, etc.) as unhealthy. We also set the average loudness of all audio data to − 25 db to keep the same recording quality among multiple types of mobile devices.

**Figure 2:**
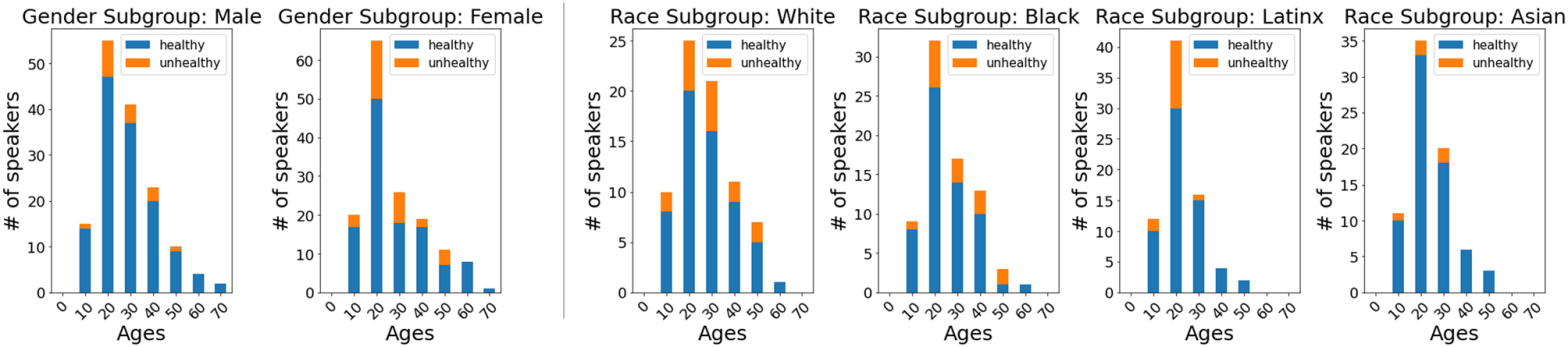
Health condition and age distribution of subgroups. Ages are positively skewed at the age of 20 years.

To explore the racial and gender disparities in voice biometrics, we set two matched datasets on race and gender, respectively. In the racial dataset, there are four sub-groups. 75 speakers with 512 snippets are collected for each sub-group. The amount of female and male speakers for White, Black, Latinx, and Asian subgroups are all 13 and 62, respectively. The average age of White, Black, Latinx, Asian are $32.02\pm 11.46$ years, $30.93 \pm 10.36$ years, $27.31 \pm 8.45$ years, $28.57 \pm 9.23$ years. The health ratio of White, Black, Latinx, Asian are 21.3%, 20.00%, 18.67%, 6.67%, respectively. Besides, in the gender dataset, there are two sub-groups, female and male. 150 female speakers and 150 male speakers are recruited, with 1444 and 1444 snippets are of female and male speakers, respectively. The amount of White, Black, Latinx, and Asian speakers for female and male subgroups are the same (104, 13, 16, and 17, respectively). The average age of females and males is $32.18 \pm 13.19$ years and $32.93 \pm 12.39$ years. The health ratio of female and male are 11.33% and 21.3%, respectively. The health condition and age distributions are shown in Fig. 2. After matching, 4936 snippets from the dataset are left, totally amounting to 411 min of ‘Aaaaah’ audio.

### Statistical analysis

We quantitatively assess the voice biometric systems by analyzing the speaker identification performance, primarily regarding the Top-1 identification accuracy. Top-1 accuracy is the rate if the classified one having the highest probability is the same as the genuine speaker. It is a standard measure of the capability of the voice biometric system and reflects the actual performance of the speaker identification in real-world applications^22^. Formally, Top-1 accuracy is defined as: $Acc=m/N$, where *m*, and *N* denote the number of corrected identification and total prediction. A higher Top-1 accuracy indicates a more outstanding speaker identification performance and better performance in voice biometrics. The results are shown in the boxplot format, where the red line represents the median, the top edge of the box is the 25% quartile, and the bottom edge of the box is the 75% quartile. The standard deviation (STD) is also employed to reflect these biometric models’ real performance further^32^, and a lower STD represents more stable speaker identification. Besides, in this work, a voice biometric model is considered to have the disparity if significant differences exist between the speaker identification performance of the subgroups, which means having privilege and disadvantage towards specific subgroups. And then, it also shows this voice biometric product contains bias. Here, we employ the significance test with one-way ANOVA and Kruskal–Wallis test (when data is non-normality and unequal variances)^33^. The outcome of the significance test is the p-value based on the dispersion correlation, which is the probability of obtaining test results at least as extreme as the results observed, assuming that the null hypothesis is correct^34^. In this work, the p-value ranges from 0 to 1, and if the p-value is less than 0.05, it indicates a significant difference among the tested data. Moreover, the 95% confidence interval (95% CI) for the true mean difference is utilized to reflect the disparity between subgroups^33^. It is a range of values that’s likely to include the true mean difference between subgroups with 95% confidence. And it indicates a significant difference between subgroups when the 95% confidence interval does not contain 0. Thereby, we disclose the disparities in voice biometric performance among racial and gender subgroups and expose the disparity source. Specifically, all statistical analyses are performed via MATLAB built-in functions^35,36^. For Voice fundamental metrics analysis, each racial subgroup contains 75 data values, each gender subgroup contains 150 data values. For voice biometrics performance, we performed k-fold cross-validation as evaluations (see “Voice biometric products” section). Each product contains 5 data values. The ANOVA and Kruskal–Wallis tests are performed across data groups.

### Voice fundamental metrics

Voice inherent characteristics are essential characteristics amid human voice, including voice intensity, pitch, duration, spectral composition, etc.^37^. They are crucial to the voice biometrics system to identify a person.

To examine if there are differences in voice inherent characteristics among different subgroups, 15 representative metrics from four different aspects of the vocal signal are utilized, representing different intrinsic properties of the human voice as shown in Table 1.

(i) Temporal: *RMS energy* is the root mean square of signal amplitude which represents continuous power of voice.
(ii) Spectral: (a) *Centroid* of spectrogram which has a robust connection with the brightness of audio; (b) *Onset* represents the number of peaks from onset strength envelope; (c) *Roll-of* is the center frequency for a spectrogram bin that at least 85% of energy is contained within this bin; (d) *Frequency of formants* (F0, F1, F2) which are local maximums of the spectrum that represents the acoustic resonance of the human vocal tract^9^.
(iii) Cepstral: MFCCs are commonly used as features in speech recognition and identification^38^. It concisely describe the overall shape of a spectral envelope. We also analyze first ($\triangle$) and second derivative ($\triangle ^{2}$) are temporal differential of MFCC that represents the rate of changes.
(iv) The voice entropy is also employed, which is a measure to describe the information capacity of a voice signal (or saying the maximum information amount can contain in a voice) and is widely considered a fundamental base of voice biometrics^16^. Four representative biometric entropy metrics are utilized: spectral entropy, PDF entropy, approximate entropy, and perm entropy. These voice entropy metrics reflect the effective information capacity by measuring different intrinsic properties of the human voice. (a) The *spectral entropy*^15^ measures the irregularity of the voice signal replying to the power spectral density of voice; (b) The *PDF entropy*^16^ measures the uniqueness and stability of the voice signal by analyzing the mutual information among voice snippets; (c) The *permutation entropy*^17^ estimates the voice complexity by capturing the order relations between the voice signal and extracting a probability distribution of the ordinal patterns; (d) The *SVD entropy*^18^ characterizes information content or regularity of a signal depending on the number of vectors attributed to the process.

**Table 1:** List of critical voice fundamental metrics.

Feature name	Voice property	Voice measurement
Spectral entropy^15^	Voice signal irregularity	The power spectral density
PDF entropy^16^	Voice uniqueness and stability	The mutual information
Permutation entropy^17^	Voice complexity	The comparison to the ordinal probability distribution
SVD entropy^18^	The complexity of voice	The dimensionality of the signal
MFCC^10^	The short-term power spectrum of voice	The shape of a spectral envelope
Formants^9^	Acoustic resonance of the vocal tract	The spectral peaks of sound spectrum
RMS^12^	Continuous power of voice	The root mean square of signal amplitude
Pitch onsets^11^	Increases in spectral energy	The number of peaks from onset strength envelope
Centroid^14^	Brightness of the voice	The centroid of spectrum
Roll-Off^13^	Approximate low bass and high treble	The center frequency for a spectrogram bin that contains $\ge 85\%$ energy

### Voice biometric products

Six state-of-the-art representative voice biometric products are utilized in this work, especially five open-source research products as shown in Table 2, which is publicly accessible^39^. (i) The speaker recognition service in *Microsoft Azure*^24^ is a commercial cloud computing service to determine a speaker’s identity from within a group of enrolled speakers. This is a publicly accessible and mature commercial product^40^. (ii) *1d-CNN* is short for the one dimension convolutional neural network. 1d-CNN takes waveform data as input and uses Mel-spectrogram-32 as the base feature, to which eight one-dimensional trainable convolutional layers are added for feature extraction. (iii) *TDNN* represents the Time Delay Neural Network^26,27^. TDNN takes Waveform data as input and uses Mel-spectrogram-32 as the base feature, to which a 5-layer deep one-dimensional trainable convolutional layer is added for feature extraction. (iv) *ResNet-18/34* are based on Residual Networks^41^. ResNet uses the preprocessed Spectrogram-257 as the base feature. Four one-dimensional convolutional layers are also added on top of the base feature. ResNet-34 (34-layer) has 16 more convolutional layers (a deeper network structure) than ResNet-18 (18-layer) in the feature extraction. (v) Different from previous open-source products, *AutoSpeech* is an automated approach to identify the optimal CNN architecture for speaker recognition^22^, rather than based on a fixed network structure. AutoSpeech uses the pre-processed Spectrogram-257 as the base feature. Several convolutional layers are added on top of the base feature. The structure of the feature extraction depends on the result of the optimization search.

Open-source research products working under the default settings are trained and tested on our matched dataset. For either exploration on racial or gender, we train these products with all subgroups based on the matched dataset (detailed in the “Matched dataset” section). It works in the same way as the application scenarios of mainstream voice biometrics products in real world. We perform 5-fold validation for each product. The training-testing ratio of splitting data for each fold is 7:2.

The assessments based on these products run on a workstation with the Linux system (Ubuntu 16.04) on an Nvidia Titan XP graphic card and an Nvidia RTX 2080 graphic card (CUDA version 10.1). We performed k-fold cross-validation as evaluations. The results from each individual fold of validation are averaged, and the standard deviation is calculated for each trial. Since each speaker has an average of five snippets, set k = 5 in this work. The advantage of this approach is that it reduces the effect of anomalous test data on the results and allows all data to be used for training and testing reflecting the actual condition.

**Table 2:** The details of open-source research products on voice biometric.

Model name	Network block	Feature
1d-CNN^25^	Convolutional layer	log Mel-spec-32 + Conv1D*8
TDNN (x-vector)^26,27^	Time-delay neural network	log Mel-spec-32 + x-vector
ResNet-18^22,29^	Residual block	Spec-257 + Featuremap [2, 2, 2, 2]
ResNet-34^22,30^	Residual block	Spec-257 + Featuremap [3, 4, 6, 3]
AutoSpeech^22^	Normal and reduction cells	Spec-257 + Searched Architecture

*log Mel-spec* log Mel-spectrogram, *Spec* spectrogram

## Results

As mentioned in “Matched dataset” section, both race and gender datasets involved in results are matched to remove the effect of unbalanced training data on the results.

**Table 3:** Voice fundamental metrics results of racial subgroups.

	White		Black		Latinx		Asian	
	Mean	STD	Mean	STD	Mean	STD	Mean	STD
Onsets	41.2012	28.2322	39.1875	28.4768	42.998	27.6175	42.0039	27.1027
RMS	0.1807	0.0619	0.1772	0.0716	0.1816	0.0689	0.1914	0.0721
Centroid	628.3296	133.7869	642.0621	165.6585	624.9091	134.0623	597.3267	118.6543
Roll-off	2261.4896	881.9737	2361.1009	968.6721	2210.6749	836.0810	2105.1559	731.9750
MFCC	$-$ 63.6165	39.9458	$-$ 63.9042	43.6219	$-$ 62.0680	38.5572	$-$ 69.1924	40.7739
$\triangle$MFCC	0.7752	1.5033	0.6574	1.3368	0.6960	1.4778	0.7030	1.4450
$\triangle ^2$MFCC	0.1147	0.3910	0.1235	0.3761	0.1023	0.3910	0.1150	0.3585
F0	134.0372	42.5300	131.9899	54.2746	132.4334	40.2225	142.1182	43.8327
F1	495.3698	158.2555	496.7421	145.8382	485.3269	165.9858	454.0798	170.0188
F2	1025.3560	218.2198	1074.4825	213.0972	1029.9507	250.5701	1008.1242	231.5954
PDF entropy	6.4428	0.0113	6.4360	0.0153	6.4395	0.0136	6.4395	0.0141
Perm entropy	1.9686	0.1622	2.0170	0.1773	1.9714	0.1701	1.9411	0.1472
Spectral entropy	9.1265	0.8457	9.2374	1.0474	9.4270	1.0227	9.2293	0.9024
SVD entropy	0.8531	0.1010	0.8577	0.1093	0.8548	0.1072	0.8254	0.1029

### Voice characteristics analysis

**Figure 3:**
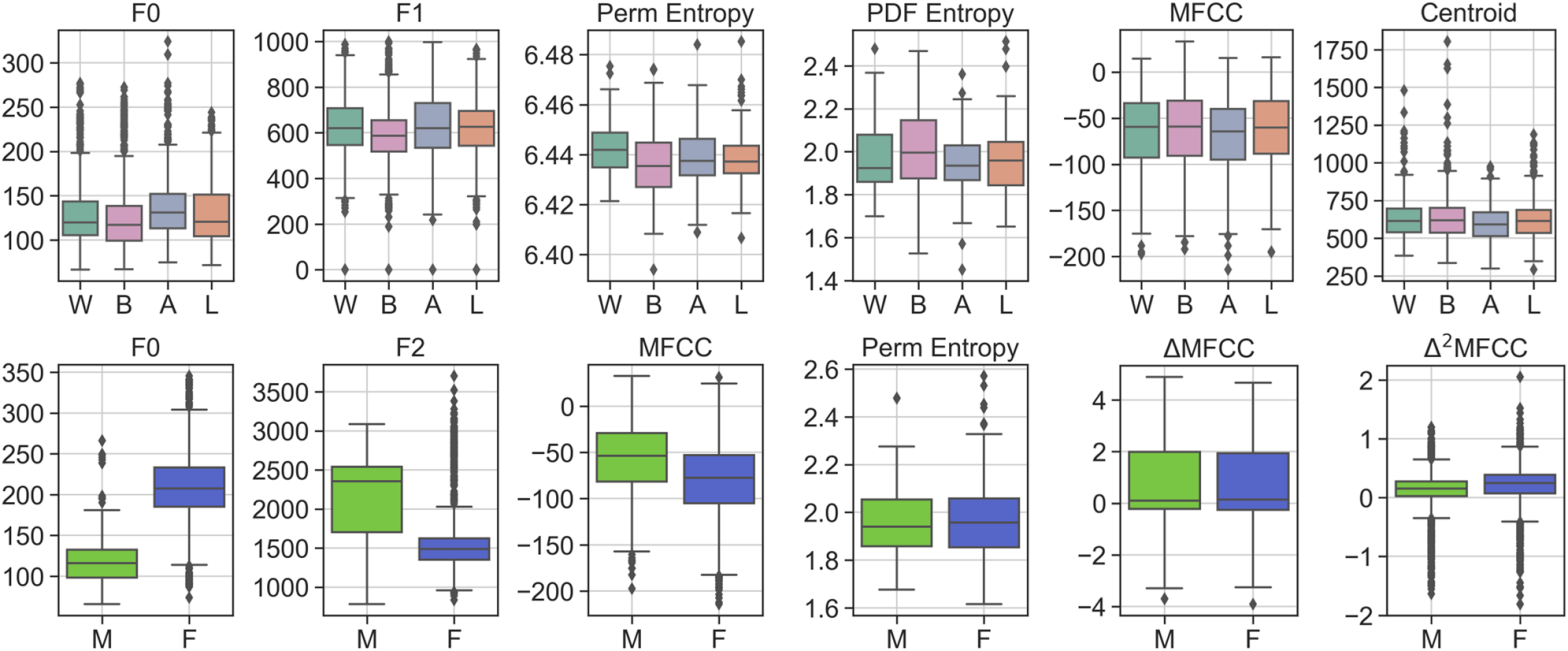
Selected voice fundamental metrics for human voice nature properties measurement on both racial and gender datasets. The y-axis is the metric value. The x-axis is subgroups (*W* White, *B* Black, *A* Asian, *L* Latinx, *M* Male, *F* Female). There are significant differences in the racial group (in F0, F1, F2, PDF entropy, and Perm entropy) and the gender group (in all metrics except $\triangle$MFCC and $\triangle ^2$MFCC).

We examine the human voice itself through a set of voice fundamental metrics. 15 representative voice fundamental metrics are utilized to measure the voice property in our matched dataset. These voice metrics reflect different nature properties of the human voice, as illustrated in the “Voice fundamental metrics” section. The box plot of voice measurements for all subgroup is shown in Fig. 3.

First, we perform the testing on the racial dataset. The results are shown in Table 3. There are no meaningful differences among most of these voice metrics, except F0, F1, F2, PDF entropy, and Perm entropy.

This means the voice properties of racial groups are similar and adequate for voice biometrics viewed from the nature voice properties in general, although slight differences exist. Then, for details, there are always performance gaps between these subgroups in some aspects.

While opposed conclusions are found on the gender dataset as shown in Table 4. Significant differences between male and female subgroups exist in various metrics. Thereby, we disclose that there significant difference in voice characteristics exists in gender subgroups but the only a slight disparity in racial subgroups.

**Table 4:** Voice fundamental metrics results of gender subgroups.

	Male		Female				
	Mean	STD	Mean	STD	p value	95% CI	
Onsets	43.91	27.73	39.78	27.70	$2.02 \times 10^{-5}$	-193.05	$-$ 71.42
RMS	0.1839	0.061	0.2130	0.076	$4.17 \times 10^{-25}$	$-$ 260.37	382.01
Centroid	599.8	120.9	667.7	150.8	$2.86 \times 10^{-42}$	361.97	483.61
Roll-off	2066	781.5	2410	905.8	$1.08 \times 10^{-29}$	290.35	411.99
MFCC	$-$ 57.86	38.60	$-$ 81.27	39.98	$2.22 \times 10^{-54}$	$-$ 542.70	$-$ 421.06
$\triangle$MFCC	0.76	1.488	0.71	1.391	0.4766	$-$ 82.90	38.73
$\triangle ^2$MFCC	0.11	0.38	0.19	0.38	$2.241 \times 10^{-18}$	210.53	332.18
F0	117.9	24.77	210.5	51.48	0	1.27	1.39
F1	522	147.12	361.4	158.8	$6.7 \times 10^{-87}$	$-$ 673.98	$-$ 552.34
F2	1048	224.4	906	184.51	$5.57 \times 10^{-110}$	$-$ 752.27	$-$ 630.62
PDF Entropy	6.440	0.011	6.448	0.014	$4.83 \times 10^{-9}$	38.99	78.25
Perm Entropy	1.966	0.157	1.972	0.171	0.8158	$-$ 17.29	21.96
Spectral Entropy	9.347	0.824	8.52	1.02	$1.79 \times 10^{-11}$	$-$ 86.96	$-$ 47.70
SVD Entropy	0.853	0.1	0.116	0.116	0.628	$-$ 24.48	14.77

### Racial disparity in speaker identification performance

**Figure 4:**
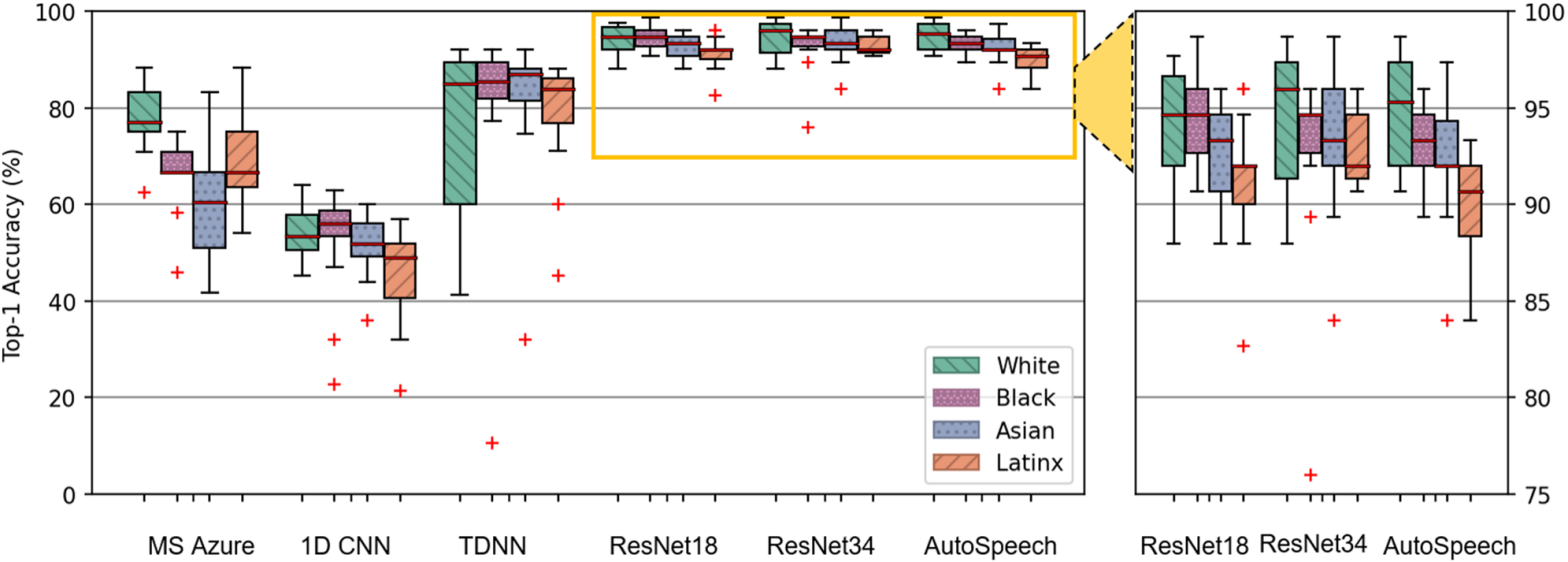
Voice identification performance among the matched racial dataset. The x-axis is racial subgroups (*W* White, *B* Black, *A* Asian, *L* Latinx). The y-axis is the percentage accuracy. ResNet-34 has the best overall accuracy. Significant differences exist among all data or between sub-groups in Microsoft Azure, 1d-CNN, ResNet-18, and AutoSpeech. No significant differences in TDNN and ResNet-34.

After investing the voice characteristics between race and gender subgroups, we explore if the speaker identification performance has a following inseparable relationship with the voice characteristics. We start by computing the Top-1 accuracy for speaker identification across our matched audio snippets within the racial dataset. For the commercial product, the commercial voice biometric model from Microsoft Azure is employed, which is a mature voice biometric product and can work on the non-speech voice. It is worth mentioning that other Tech Giants or companies (e.g., Apple, IBM, Google, Amazon, Facebook) do not have publicly accessible commercial voice biometrics or speaker recognition products on non-speech audio. We also note that since the speaker recognition service of Microsoft Azure (Version 1.14.0, March 2021) is limited to 24 users, therefore, we randomly select 24 different speakers (12 females and 12 males) from the matched dataset when evaluating the racial disparity, and 24 different speakers (six Whites, six Blacks, six Latinxs, six Asians) when examining the gender disparity. Both these selected datasets are matched. Other open-source biometric models follow the complete matched datasets. The identification results are shown in Fig. 4. The average Top-1 accuracy for White speakers is 77.58% (STD 7.53%) higher than with Latinx (69.67%, STD 9.81%), black (65.83%, STD 8.29%), Asian (61.22%, STD 13.13%) speakers, respectively. A significant difference exists among all subgroups on the whole (p < 0.01), and the performance on White speakers is outstandingly higher than Black and Asian subgroups (p < 0.01, 95% CI 4.31, 19.19; p < 0.01, 95% CI 6.31, 26.42), respectively. Since this commercial product is black-box (the details about this voice biometric model or other related knowledge are not accessible to the public) and contains the racial disparity in the related application (the related functionality (speech recognition) in cognitive services from Microsoft Azure has been reported containing racial bias^7^) due to unbalanced training data samples, we continue exploring the racial disparity with the following representative open-source models.

For the open-source products, we build the speaker identification systems from the sketch under the default settings from its original paper based on our matched dataset. There are mainly two types of voice biometric products. Except for the neural network-based type mentioned in the following, the statistic-based type for voice biometric products (e.g., i-Vector and GMM-UBM) primarily utilizes the phonemes (pitch, cadence, and inflection) for speaker identification. However, The ‘Aaaaah’ utterances are too short to meet statistic-based voice biometric products’ requirements. The statistic-based voice biometric products at least need 20–80 unique phonemes with a duration of 1–2 min, which does not apply to the current dataset^21^. For five state-of-the-art neural network-based voice biometric products, Fig. 4 shows that the identification performances of subgroups are all different in these products. For example, for the ResNet-34 model, which has the best overall performance, the Top-1 accuracy for White speakers is 97.33% (STD 4.09%) compared with Asians 94.67% (STD 3.62%), Blacks 90.67% (STD 4.91%), and Latinxs 88.00% (STD 2.34%), respectively. However, for the AutoSpeech model that also has an excellent overall performance, the Black subgroup (90.67%, STD 2.42%) has a better performance than Asian (88.00%, STD 3.71%), and Latinx speakers (85.33%, STD 2.83%), respectively, although the White subgroup still gets the best performance with 97.33% (STD 3.23%). The results illustrate that no particular racial group has the best performance over others among all these speaker identification models, and no specific racial group always has the worst performance. Besides, significant performance gaps are uncovered between these subgroups. In the CNN model, the performances from all subgroups are significantly different in general (p = 0.02), and the White and Black subgroups are remarkably better than the Latinx subgroup (p = 0.01, 95% CI 1.58, 14.15; p < 0.01, 95% CI 2.25, 14.81), respectively. In the AutoSpeech model, a significant difference exists among all subgroups on the whole (p = 0.02), and the White and Black subgroups are outstandingly better than the Latinx subgroup (p = 0.03, 95% CI 1.95,7.66; p = 0.02, 95% CI 1.12, 11.28), respectively. In the ResNet-18 model, the White subgroup is extraordinarily better than the Latinx subgroup (p = 0.02, 95% CI 1.16, 13.50). These indicate that both commercial and open-source voice biometric models exist disparities among these racial subgroups.

### Gender disparity in speaker identification performance

**Figure 5:**
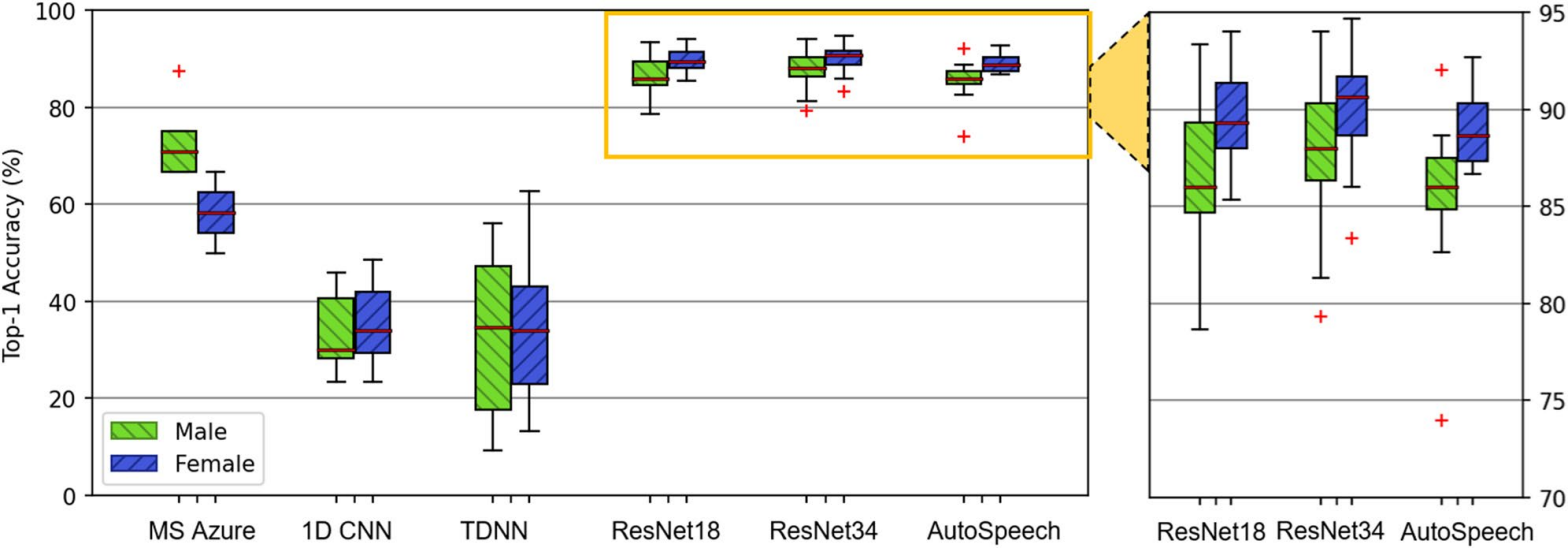
Voice identification performance among the matched gender dataset. The x-axis is gender subgroups. The y-axis is the percentage accuracy. ResNet-34 has the best overall accuracy. Significant differences between females and males are discovered in Microsoft Azure, ResNet-18, ResNet-34, and AutoSpeech. No significant differences in TDNN and 1d-CNN.

We continue exploring the disparity on the matched gender dataset. For the commercial product from Microsoft Azure, the average Top-1 accuracy for male speakers are 73.33% (STD 8.64%), and for female speakers are 58.33% (STD 6.59%). The performance of male speakers is significantly higher than female speakers (p = 0.01, 95% CI − 26.20, − 3.80), demonstrating gender disparity exists in the Microsoft Azure speaker recognition service. Besides, integrated with^7^, this result further reveals that the racial disparity widely exists in almost all of the cognitive services related to the speech on Microsoft Azure as a result of unbalanced training data samples on the whole platform. For state-of-the-art open-source voice biometric products, Fig. 5 shows that among all these speaker identification models, the performance of female speakers is better than male speakers. For example, for the ResNet34 model with the best overall performance, the average Top-1 accuracy for female speakers is 92.00% (STD 2.97%) compared with 90.67% (STD 3.94%) for male speakers. Also, in the AutoSpeech model with excellent overall performance, the female subgroup gets the better performance with 89.00% (STD 2.18%), which contrasts to 85.34% (STD 4.71%) for male speakers. These results indicate these neural network-based models may have the same preference on the gender subgroups. Besides, significant performance gaps are revealed between these subgroups in some biometric models. In the ResNet-18, ResNet-34, and AutoSpeech model, the female subgroup is all significantly superior to the male subgroup (p = 0.01, 95% CI 0.83, 5.23; p = 0.03, 95% CI 0.28, 4.75; p = 0.02, 95% CI 1.23, 11.57). This indicates that the voice biometric models, no matter the commercial models or open-source models, exist disparities among these gender subgroups.

## Result analysis and discussion

### Study on causal factors

There are two representative categories that could account for these racial and gender disparities in the voice biometrics domain, (i) the voice characteristic cause: since different races or genders produce the voice with different properties, it is natural to wonder if the general nature properties (e.g., phonation) of the human voice itself limit the speaker identification performance, and (ii) the technical cause: there are two main components in the voice biometric system: feature extraction and classification. Feature extraction extracts specific characteristics from the original voice snippets, and classification is to verify the user identity based on these learned characteristics. Thus, another important concern is if the technology in the voice biometric model prohibits individual identification (e.g., limited feature selection)^42,43^. The results in the “Voice characteristics analysis” section indicates there is a slight difference between voice for racial subgroups (e.g., in F1). Moreover, there were significant differences between gender subgroups among 15 voice fundamental metrics. Therefore, we investigate causal factors for race and gender separately.

#### Racial causal factors

The results in the “Voice characteristics analysis” section indicate the radical disparities in voice biometrics are not predominantly from the voice itself since most of the voice fundamental measures do not differ between racial groups. We further scrutinize *the technical cause*, the biometric technology itself. Due to requirements for the computing time and recourse, these features can only reflect some principal properties of the human voice (not all properties), which can unwittingly amplify the racial disparities in the final voice biometrics outcomes.

#### Gender causal factors

Since there are significant differences in voice characteristics in the gender subgroups, we hypothesize that the gender disparities are primarily caused by both voice inherent properties and limited feature extraction.

**Figure 6:**
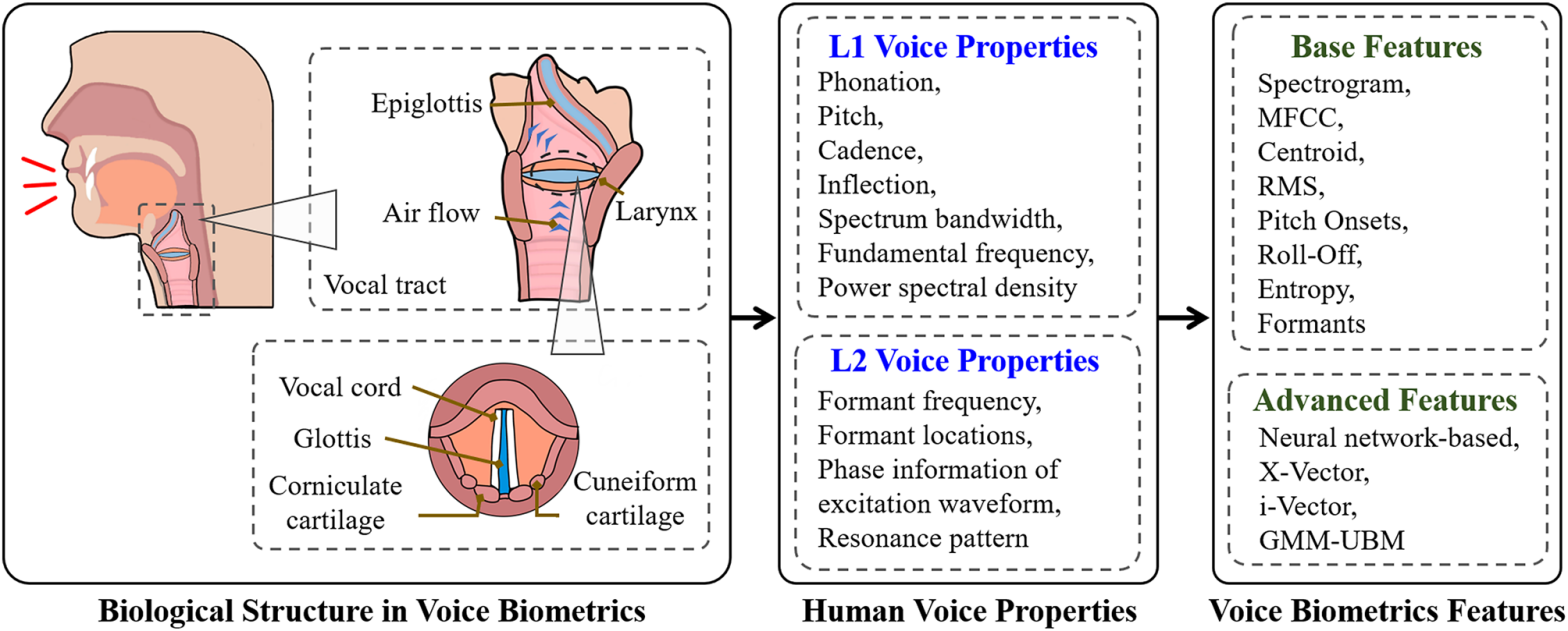
A taxonomy of the vocal biological structure, voice properties, and computational voice features for voice biometrics.

### Disparities sources detection

As shown in Table 1, a list of critical voice fundamental metrics/features are recruited to measure voice properties from different perspectives (e.g., temporal, spectral, and cepstral), which can reflect the voice characteristics of the speaker and aid us to interpret the results of the voice biometric products. To understand the voice features utilized by voice biometric products, we first explore the relation between vocal biological structures and dominant voice inherent characteristics used for speaker identification, as shown in Fig. 6. There are two levels of voice inherent characteristics/properties utilized in voice biometric products. The first level is based on L1 voice properties, the general characteristics of the voice (e.g., phonation). Phonation is the process by which the vocal folds produce certain sounds through quasi-periodic vibration, which also depends on the activity of the muscles and the position of the cartilages of the larynx^44^. The second level utilizes L2 voice properties, the minutiae points (e.g., formant frequency, formant locations).

The formant is the distinctive frequency component of the acoustic signal and is usually defined as a broad peak, or local maximum, in the spectrum. The formants are highly determined by the length of the vocal tract and vocal fold. We can assess the acoustic resonance of the vocal tract by searching spectral peaks of the sound spectrum. The formant with the lowest frequency is called F1, and then the second F2. Most often, the two formants, F1 and F2, are fundamental and crucial characteristics in the human voice, including non-speech and speech voice^45,46^.

As discussed in the “Voice characteristics analysis” section, for racial subgroups, the principal differences in voice properties result from phonation/formant. Latinx vowels (including /ɑ/) are generally shorter (in duration) than other subgroups, vary little in quality and remain contrastive in stressed and unstressed positions^47^. Moreover, Latinx speakers have lower F1 during isolated /ɑ/ prolongations compared to White speakers. Besides, for gender subgroups, male speakers have longer vocal tract and vocal fold dimensions and lower formants centralized within the low-frequency band on the spectrum^48,49^. After examining this taxonomy for voice biometrics, we continue to discover how the technology prohibits individual identification.

**Figure 7:**
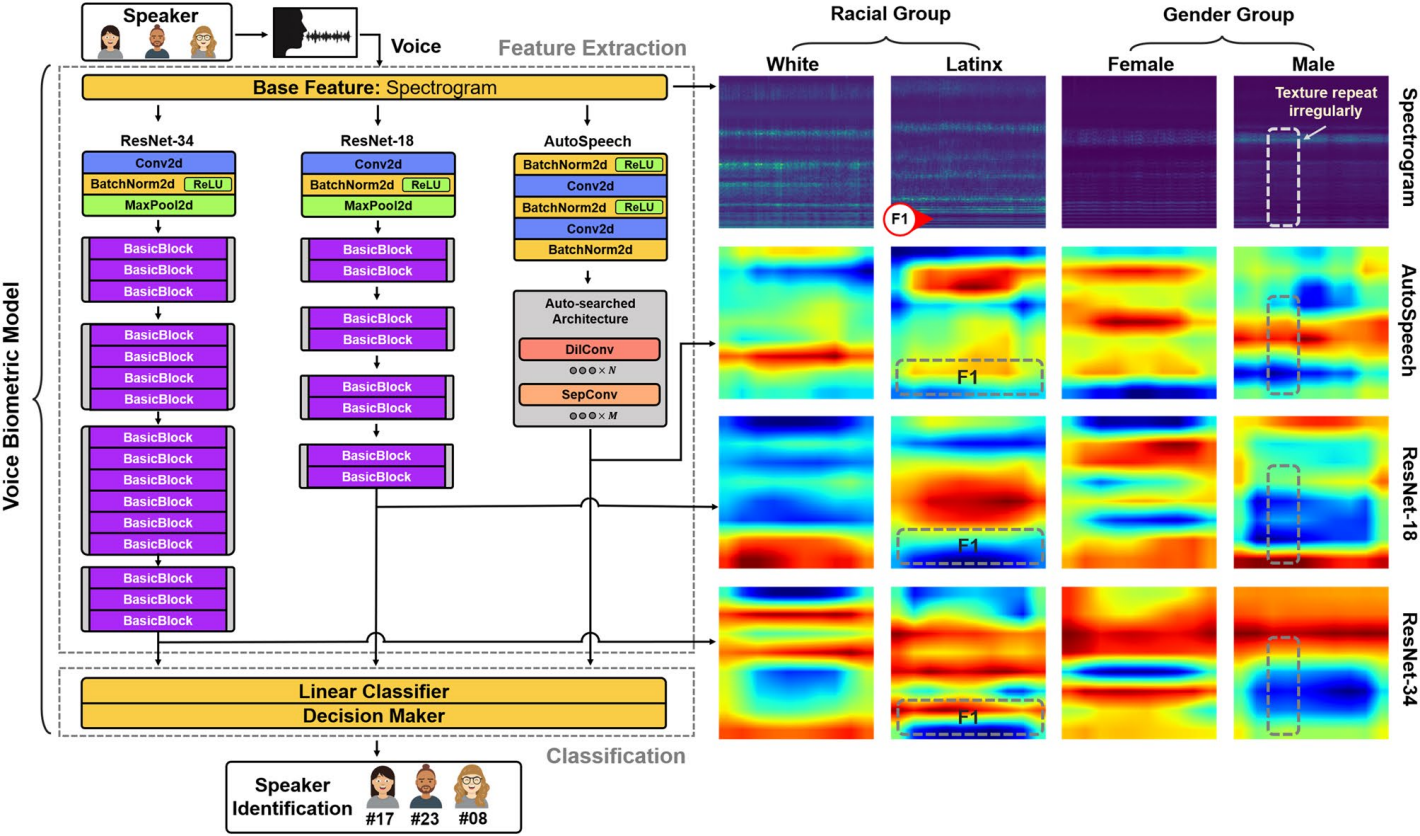
The voice biometric system is mainly divided into two parts: feature extraction and classification. The feature extraction part includes the base feature and neural network-based feature maps. ResNet-18, ResNet-34, and AutoSpeech utilize the same classification and base feature (spectrogram). The racial disparity is discovered in ResNet-18 and AutoSpeech, and gender disparity is detected in these three. The neural network-based feature extraction primarily causes these disparities. F1 is noted for the first format here. The average F1 for White speakers is 495.3 Hz, for Latinx speakers is 485.3 Hz.

To further disclose the source of these racial and gender disparities, we examine the learned voice characteristics/properties in the feature extraction outputs of these products. The feature extraction usually includes the base features and neural network-based feature maps. Considering that ResNet-18, ResNet-34, and AutoSpeech use the same classifier and base feature (spectrogram) and have different preferences on our matched datasets mentioned above, the voice characteristics (feature weights) learned from these three models are shown in Fig. 7. The results show learned features within these three biometric models mostly weigh on the voice properties related to formants^50^. In the racial group, Latinx speakers have lower F1 during isolated/ɑ/prolongations than White speakers^51^, making these feature extractions more difficult to locate the F1 band based on convolution layer-based solutions. Consequently, the feature extractions are limited to extracting the useful voice characteristics from Latin speakers in both ResNet-18 and AutoSpeech models, which jeopardizes the final speaker identification decision and causes racial disparity. Besides, in the gender group, the males’ formats are mainly located on the low-frequency area, and subsequently, the texture on the males’ spectrogram repeats more irregularly compared to females’. Since the classic convolutional kernel utilized in these products is less effective in generalizing such irregular patterns due to shape mismatch^52^, the neural network-based feature extraction is restricted to further unearth the voice identity on these three models^53,54^. Moreover, similar situations can be observed in the rest research voice biometric models. Thereby, we further discover that racial disparities primarily result from the neural network-based feature extraction within the voice biometric product and gender disparities primarily due to both voice inherent characteristic difference and neural network-based feature extraction.

### Disparities discussion

As noted above, our findings indicate that the overall racial subgroup has a slight difference in voice inherent characteristics (e.g., in F1), and differences in genders subgroups are gigantic. Disparities exist between both racial and gender groups in several biometric products (e.g., ResNet-18, AutoSpeech) towards particular subgroups (e.g., Latinxs). We identify racial disparities in voice biometrics are not primarily related to the voice characteristics, but from the technical cause, a gap in the feature extraction. On the other hand, gender disparities are primarily related to the voice inherent characteristics and the feature extraction technology. The results indicate that the neural network-based feature extractions are limited in learning the comprehensive voice characteristics for voice biometrics to some extent^55^.

Currently, AutoSpeech is widely recognized to achieve the highest speaker identification performance among the open-source research products (noted on July 2021: AutoSpeech achieves the best performance of speaker identification on VoxCeleb1 verified by Paperwithcode), but it has perceptible racial and gender disparities. Our findings reveal that when designing the voice biometric product, rather than only focusing on the entire performance of the representative organized voice dataset (e.g., VoxCeleb1^56^), we also need to pay attention to the subgroups’ performance. Besides, to improve the speaker identification performance or mitigate disparities, feature extraction optimization is also an option^57,58^, more than just working on the classifier.

Deep features are high-level features that are automatically learned by the deep neural network through the data in several iterations. The understanding and interpretation of deep features is still a challenge, so manual intervention to avoid model bias toward demographic backgrounds is very difficult. Therefore, to overcome this problem, our system can be used as a tool for evaluating voice biometric products, quantifying the fairness of the voice biometric model through matched datasets. Moreover, it can provide indications for multi-model fusion to reduce voice biometrics product bias.

In our study, the speakers collected in our matched datasets are from 15–70 years in each subgroup. Most speakers are in the generation of 20–40 years. Nevertheless, it is possible that at least some of the differences we see are mainly a result within the 20–40 years generation, not all ages. This does not revoke the discovery of radical and gender disparities in voice biometric models. We hope to extend the future work by examining the voice biometrics performance among speakers from other generations.

Furthermore, it is time for related researchers, engineers, investors, and governors to rethink this technology comprehensively to ensure that it has a low possibility of causing potential hazards or bias toward particular subgroups. Besides, another problem we need to care about is to prevent such disparities affecting the prevailing cultural, social norms, or legal regulations and to avoid secondary victimization.

## Data Availability

The data that support the findings of this study are available from the corresponding author upon reasonable request. The data are not publicly available because they contain information that could compromise research participant privacy/consent.
